# A prognostic model for hepatocellular carcinoma based on apoptosis-related genes

**DOI:** 10.1186/s12957-021-02175-9

**Published:** 2021-03-12

**Authors:** Renjie Liu, Guifu Wang, Chi Zhang, Dousheng Bai

**Affiliations:** 1 Department of Hepatobiliary Surgery, Clinical Medical College, Yangzhou University, Yangzhou, 225009 Jiangsu People’s Republic of China; 2 Dalian Medical University, Dalian, 116044 Liaoning People’s Republic of China

**Keywords:** Apoptosis-related genes, Hepatocellular carcinoma, Prognostic model, The Cancer Genome Atlas, Nomogram

## Abstract

**Background:**

Dysregulation of the balance between proliferation and apoptosis is the basis for human hepatocarcinogenesis. In many malignant tumors, such as hepatocellular carcinoma (HCC), there is a correlation between apoptotic dysregulation and poor prognosis. However, the prognostic values of apoptosis-related genes (ARGs) in HCC have not been elucidated.

**Methods:**

To screen for differentially expressed ARGs, the expression levels of 161 ARGs from The Cancer Genome Atlas (TCGA) database (https://cancergenome.nih.gov/) were analyzed. Gene Ontology (GO) enrichment and the Kyoto Encyclopedia of Genes and Genomes (KEGG) pathway analyses were performed to evaluate the underlying molecular mechanisms of differentially expressed ARGs in HCC. The prognostic values of ARGs were established using Cox regression, and subsequently, a prognostic risk model for scoring patients was developed. Kaplan–Meier (K-M) and receiver operating characteristic (ROC) curves were plotted to determine the prognostic value of the model.

**Results:**

Compared with normal tissues, 43 highly upregulated and 8 downregulated ARGs in HCC tissues were screened. GO analysis results revealed that these 51 genes are indeed related to the apoptosis function. KEGG analysis revealed that these 51 genes were correlated with MAPK, P53, TNF, and PI3K-AKT signaling pathways, while Cox regression revealed that 5 ARGs (PPP2R5B, SQSTM1, TOP2A, BMF, and LGALS3) were associated with prognosis and were, therefore, obtained to develop the prognostic model. Based on the median risk scores, patients were categorized into high-risk and low-risk groups. Patients in the low-risk groups exhibited significantly elevated 2-year or 5-year survival probabilities (*p* < 0.0001). The risk model had a better clinical potency than the other clinical characteristics, with the area under the ROC curve (AUC = 0.741). The prognosis of HCC patients was established from a plotted nomogram.

**Conclusion:**

Based on the differential expression of ARGs, we established a novel risk model for predicting HCC prognosis. This model can also be used to inform the individualized treatment of HCC patients.

**Supplementary Information:**

The online version contains supplementary material available at 10.1186/s12957-021-02175-9.

## Introduction

Globally, liver cancer is the sixth most common tumor and the fourth leading cause of cancer-associated mortalities [1]. Among the frequent primary liver cancers, hepatocellular carcinoma (HCC) accounts for approximately 75% of all cases [1]. Despite advances in diagnostic techniques and treatment, HCC is still associated with poor survival outcomes due to the high rate of recurrence and metastasis [2–4]. The TNM staging system is a traditional method for prognostic prediction; however, it lacks performance accuracy due to substantive variations in HCC clinical outcomes [5]. Over the past decades, serum alpha-fetoprotein (AFP) has been the only biomarker for detecting and predicting the prognosis of HCC; however, its low sensitivity limits its clinical utility [6]. Therefore, the identification of a novel prognostic biomarker and establishment of an advanced prognostic model for HCC patients is of paramount importance.

Bioinformatics analysis is important in elucidating the functions of numerous differentially expressed genes as well as evaluating the complexity of HCC occurrence and development [7, 8]. Meng et al. used a series of bioinformatics analyses to identify hub genes and pathways associated with HCC pathogenesis and prognosis [9]. However, these studies usually ignore clinical information such as sex, age, grade, and stage of tumors. It may be very innovative, and informative to construct a prognostic model that combines patient’s gene expression level and clinical information. Hepatocarcinogenesis develops following an imbalance between proliferation and apoptosis [10]. It has been documented that the overexpression of spindle and kinetochore-related complex subunit 3 (SKAT3) in HCC inhibits P53 activation by binding cyclin-dependent kinase 2 (CDK2), before impeding cell apoptosis, and thereby promoting cancer cell proliferation [11]. Besides, several biomolecules may influence HCC prognosis by regulating apoptosis-related genes (ARGs) or apoptosis-related pathways [12–14]. Yu et al. evaluated the association between the haplotype of the apoptosis-related gene cyclin-dependent kinase inhibitor 1B (CDKN1B) and the prognosis of HCC patients who were subjected to surgical resection [15]. The CCT/ACT haplotype patients were found to exhibit lower overall survival rates than those with the more common ACT/CCT haplotype. Therefore, ARGs can potentially be used to assess HCC prognosis.

Based on the gene expression and clinical characteristics data obtained from the Cancer Genome Atlas (TCGA) database, we established the ARGs associated with HCC prognosis and developed a prognostic prediction model. The model calculates the risk score to predict and evaluate the HCC prognosis.

## Materials and methods

### Data collection

The mRNA expression data and clinical information of HCC patients were obtained from the TCGA database (https://cancergenome.nih.gov/). The obtained clinical information included age, gender, TNM stage, T stage, N stage, M stage, and histological grade. A total of 161 ARGs were acquired from the gene set “HALLMARK_APOPTOSIS” in the Molecular Signatures Database v7.1 in GSEA [16].

### Gene set enrichment analysis and differentially expressed ARGs

Gene sets with significant differences between HCC and normal samples were evaluated by GSEA. Subsequently, using the mRNA expression profiles, the limma package and the Wilcoxon signed-rank test in R software 3.6.2 (|log_2_FC| > 1, FDR < 0.05) were used to show the significantly differently expressed ARGs in the HCC cohort. The pheatmap and ggpubr packages in R software were used to develop volcano plots, heatmaps, and box plots.

### Functional enrichment, KEGG pathway, and PPI network analysis

Gene ontology (GO) enrichment and the Kyoto Encyclopedia of Genes and Genomes (KEGG) pathway analysis were performed to evaluate the potential biological functions of ARGs, after which they were visualized through R software packages such as ggplot2, DOSE, clusterProfiler, enrichplot, GOplot, digest, etc. Interactions among the selected ARGs were determined through protein–protein interaction (PPI) networks from the STRING database (http://www.string-db.org/) [17] and visualized by Cytoscape software [18].

### Establishment of a prognostic risk model based on ARGs

Univariate and multivariate Cox proportional hazard regression analyses were performed to identify prognosis-associated ARGs in HCC. Then, a prognosis-associated prediction formula, acquired from multivariate Cox regression analysis, was used to construct a prognostic model using the “glmnet” package in R. Using the prognostic model, Kaplan–Meier (K-M) analysis was performed to evaluate the survival rates of the high- and low-risk groups. Subsequently, the area under the receiver operating characteristic (ROC) curve (AUC), and KEGG enrichment analysis were used to assess the predictive value of the prognostic model. Finally, the R package (rms) was used to develop a risk model-based nomogram for predicting the prognosis of HCC patients.

### Statistical analysis

The gene expression data were normalized by log2 transformation. Thereafter, statistical analyses were performed, while plots were constructed using the R software 3.6.2 (https://www.r-project.org/) and Perl language packages. *p* ≤ 0.05 was considered statistically significant.

## Results

### Acquisition of apoptosis-related genes set and GSE analysis

**Fig. 1:**
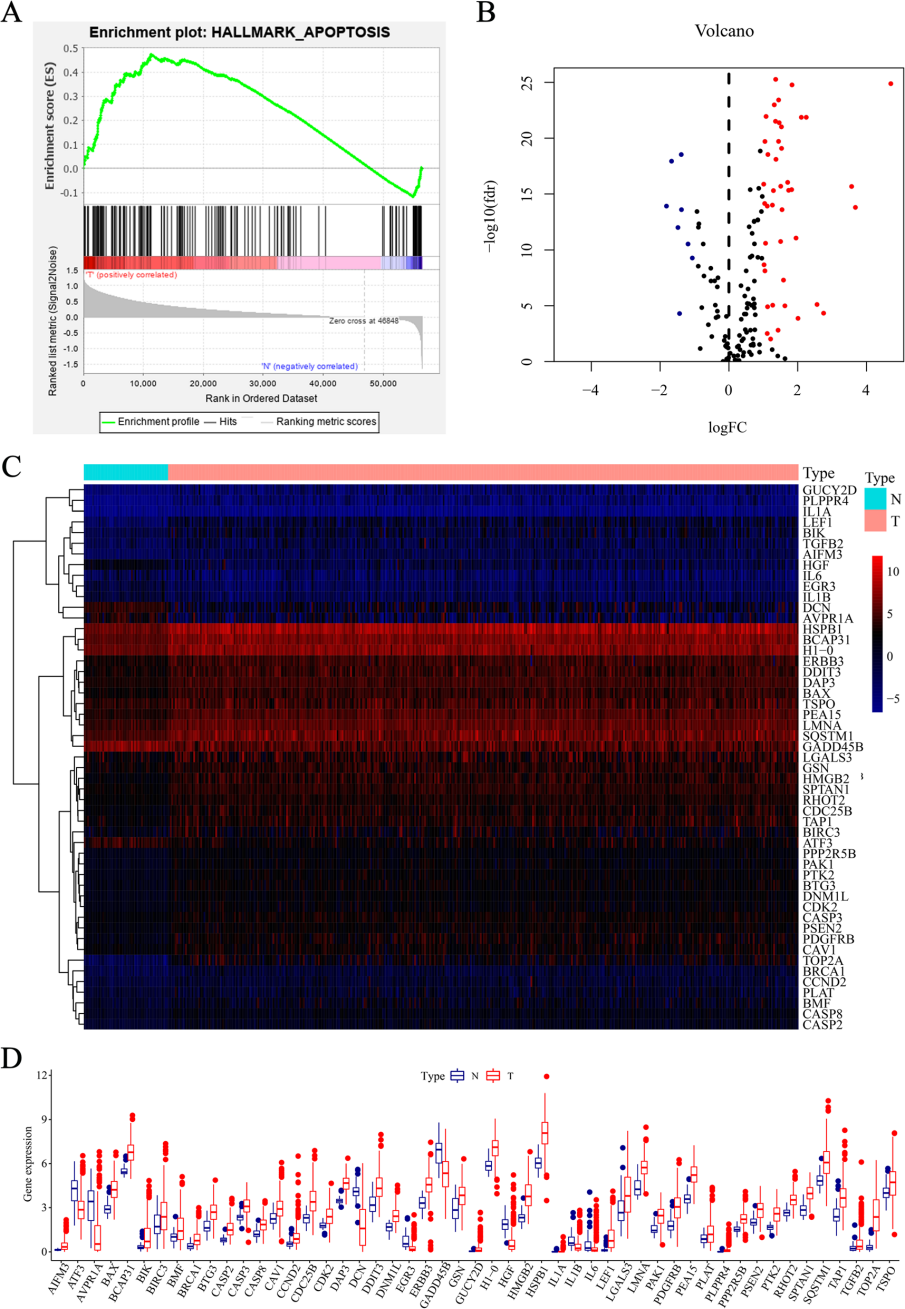
Identification of differentially expressed 51 apoptosis-related genes (ARGs) in HCC. **a** GSE analysis of 161 ARGs. **b** Volcano plot of differentially expressed ARGs. Red represents high expression, blue represents low expression, black represents no difference between HCC and normal tissues. **c** The heatmap of 51 identified ARGs. **d** The boxplot of 51 identified ARGs. Red represents HCC tissues, while blue represents normal tissues, respectively. *ARGs* apoptosis-related genes. *HCC* hepatocellular carcinoma

Following the search in the Molecular Signatures Database v7.1 in GSEA, 161 ARGs were identified from the gene set “HALLMARK_APOPTOSIS”, which were listed in Additional file 1. Thereafter, the 161 ARGs in this gene set were further assessed through GSEA analysis to ascertain their biological significance in HCC. Figure 1a shows that ARG set was significantly differentially expressed between HCC and normal samples.

### Identification of differentially expressed ARGs

The mRNA sequence data for 374 HCC tissue samples and 50 samples of normal tissue were obtained from the TCGA database. The limma package and the Wilcoxon signed-rank test in R (|log_2_FC| > 1, FDR < 0.05) were used to screen differentially expressed ARGs in HCC and non-tumor samples. In this study, 43 and 8 ARGs were found to be significantly upregulated and downregulated, respectively. These findings are presented in a volcano plot, heatmap, and box plot (Fig. 1b–d).

### Functional enrichment and PPI network analysis of differentially expressed ARGs

**Fig. 2:**
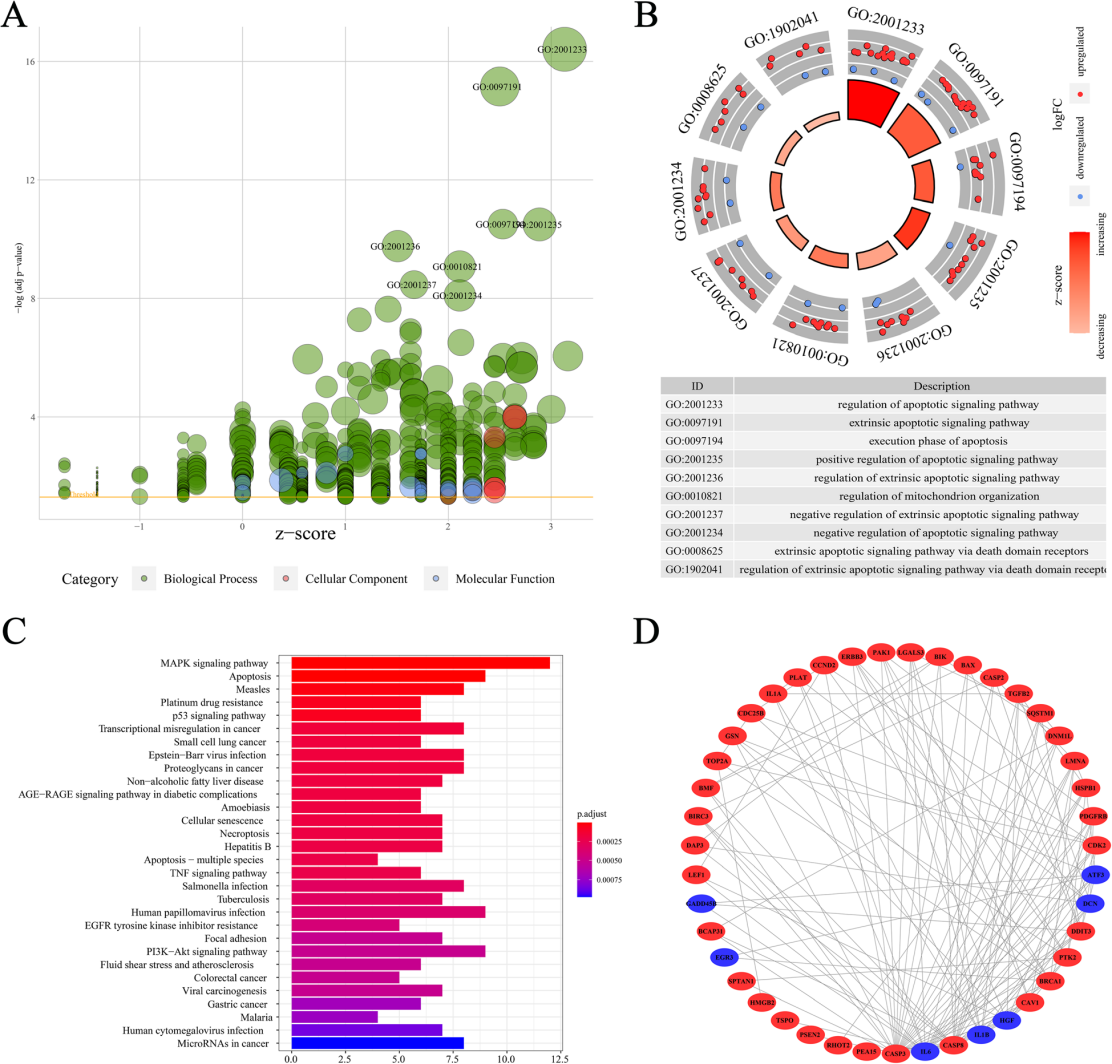
GO enrichment, KEGG pathway, and PPI analysis of the 51 identified ARGs. Findings of the GO analysis are presented in the bubble chart (**a**) and the circle plot (**b**). **c** Results from KEGG analysis are presented in the bar plot. **d** The PPI network of the 51 identified ARGs

To establish the biological functions and significant pathways of the 51 identified genes, GO enrichment and KEGG pathway enrichment analysis were performed (Fig. 2). The 51 identified ARGs were found to be involved in the pathways associated with cellular apoptosis (Fig. 2a and b). Furthermore, KEGG enrichment analysis revealed that these ARGs are involved in platinum drug resistance, transcriptional dysregulation in cancer, and some oncogenic pathways such as MAPK, P53, TNF, and PI3K-AKT signaling pathways (Fig. 2c). The STRING online tool was used to establish a PPI network to evaluate the interactions among ARG-coded proteins. Results were observed using the Cytoscape software (Fig. 2d).

### Association between ARGs and HCC patient’s survival and prognosis

**Fig. 3:**
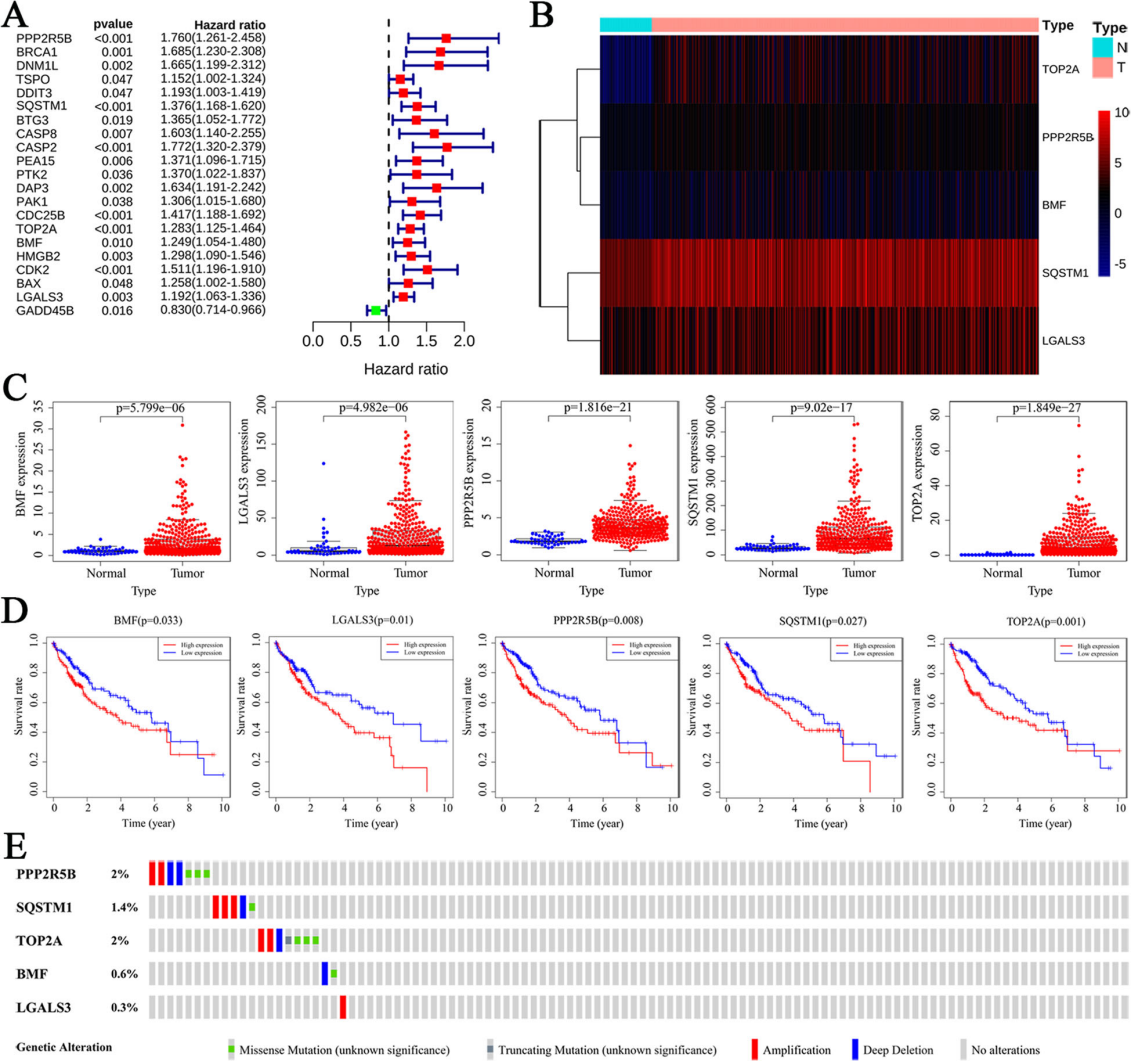
Identification of ARGs associated with the prognosis of HCC. **a** The forest plot of univariate Cox regression results. **b** The heatmap of 5 identified prognostic ARGs screened out by multivariate Cox regression. **c** Expression levels of 5 screened ARGs in HCC and normal samples. **d** K-M curve of the relationship between OS in HCC patients and expression levels of 5 screened ARGs. **e** Mutation data of 5 screened ARGs among 353 HCC specimens according to the cBioPortal database. *OS* overall survival rates

Univariate Cox regression analyses were performed on both mRNA expression and the corresponding clinical data for the 51 selected ARGs to identify their prognosis-associated ARGs in HCC (Fig. 3a). There were 20 upregulated ARGs and 1 downregulated ARG which were statistically significant. These 21 genes were subsequently analyzed by multivariate Cox regression (*p* < 0.05) to determine their association with the prognosis of HCC patients and to acquire the corresponding regression coefficients. Five prognosis-related ARGs: Protein phosphatase 2 regulatory subunit B'beta (PPP2R5B), Sequestosome 1 (SQSTM1), DNA topoisomerase II alpha (TOP2A), Bcl2 modifying factor (BMF), and Galectin 3 (LGALS3) were screened. Expression levels in normal and HCC samples were further compared to establish a prognostic value involving these 5 genes. Compared to normal samples, the 5 identified ARGs in HCC samples exhibited significantly elevated expression levels than normal specimens as indicated by heatmap and boxplot (Fig. 3b and c). Besides, the K-M curve was constructed by utilizing the survival rate differences in the high- and low-expressed groups of the identified ARGs. Interestingly, the high expression levels of the 5 identified ARGs indicated a low survival rate (Fig. 3d).

Furthermore, mutations in these 5 HCC genes were analyzed through the cBioPortal database (http://cbioportal.org). Data from 353 HCC patients in this database revealed that 22 patients (6.3%) had mutations. Among the 22 patients with the mutation, 0.85% had missense mutations, 0.56% had amplifications, and 0.56% had deep deletions in the PPP2R5B gene; 0.28% had missense mutations, 0.85% had amplifications, and 0.28% had deep deletions in the SQSTM1 gene; while 0.85% had missense mutations, 0.28% had truncating mutations, 0.56% had amplifications, and 0.28% had deep deletions in the TOP2A gene. Moreover, 0.28% had missense mutations, and 0.28% had deep deletions in the BMF gene whereas 0.28% had amplifications in the LGALS3 gene (Fig. 3e).

**Fig. 4:**
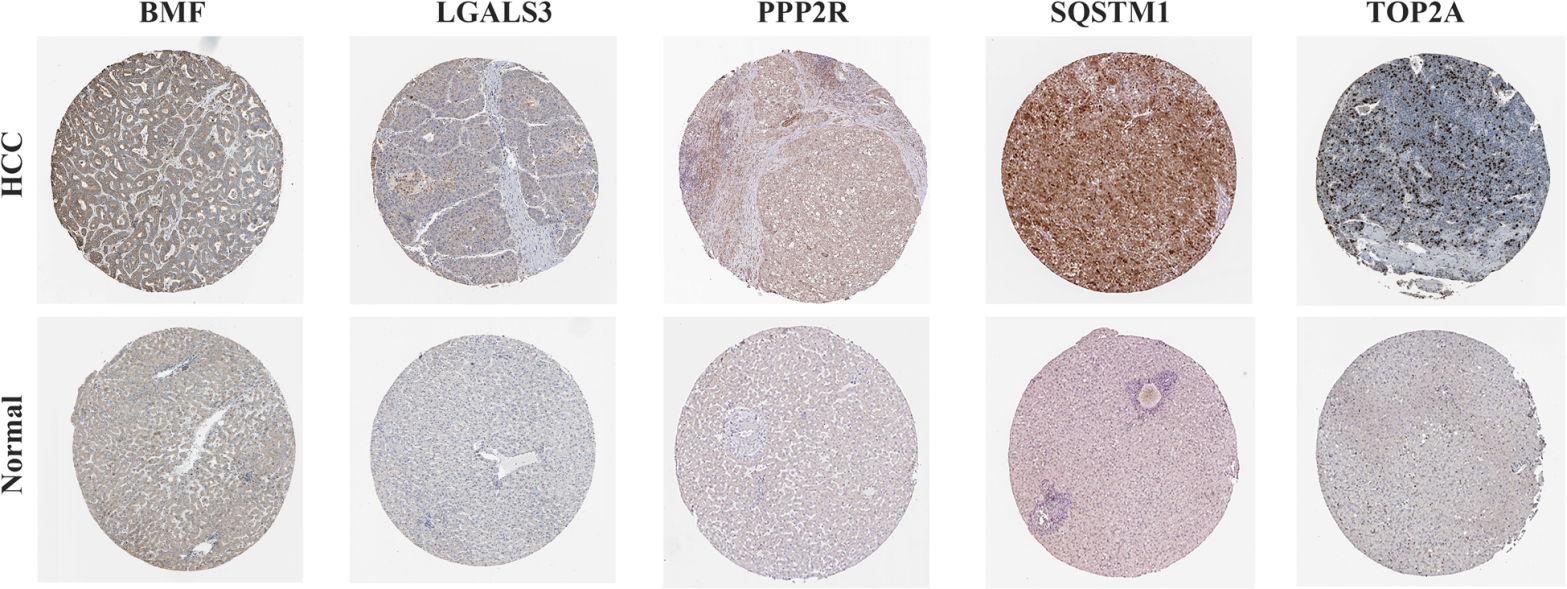
Boxplot and immunohistochemical results of the expression levels of 5 screened ARGs between HCC and para-carcinoma tissues according to the Human Protein Atlas (HPA) database

Immunohistochemical analysis of the Human Protein Atlas (HPA) database revealed that these 5 genes were significantly upregulated in HCC (Fig. 4).

### Establishment of a prognostic risk signature based on ARGs

**Fig. 5:**
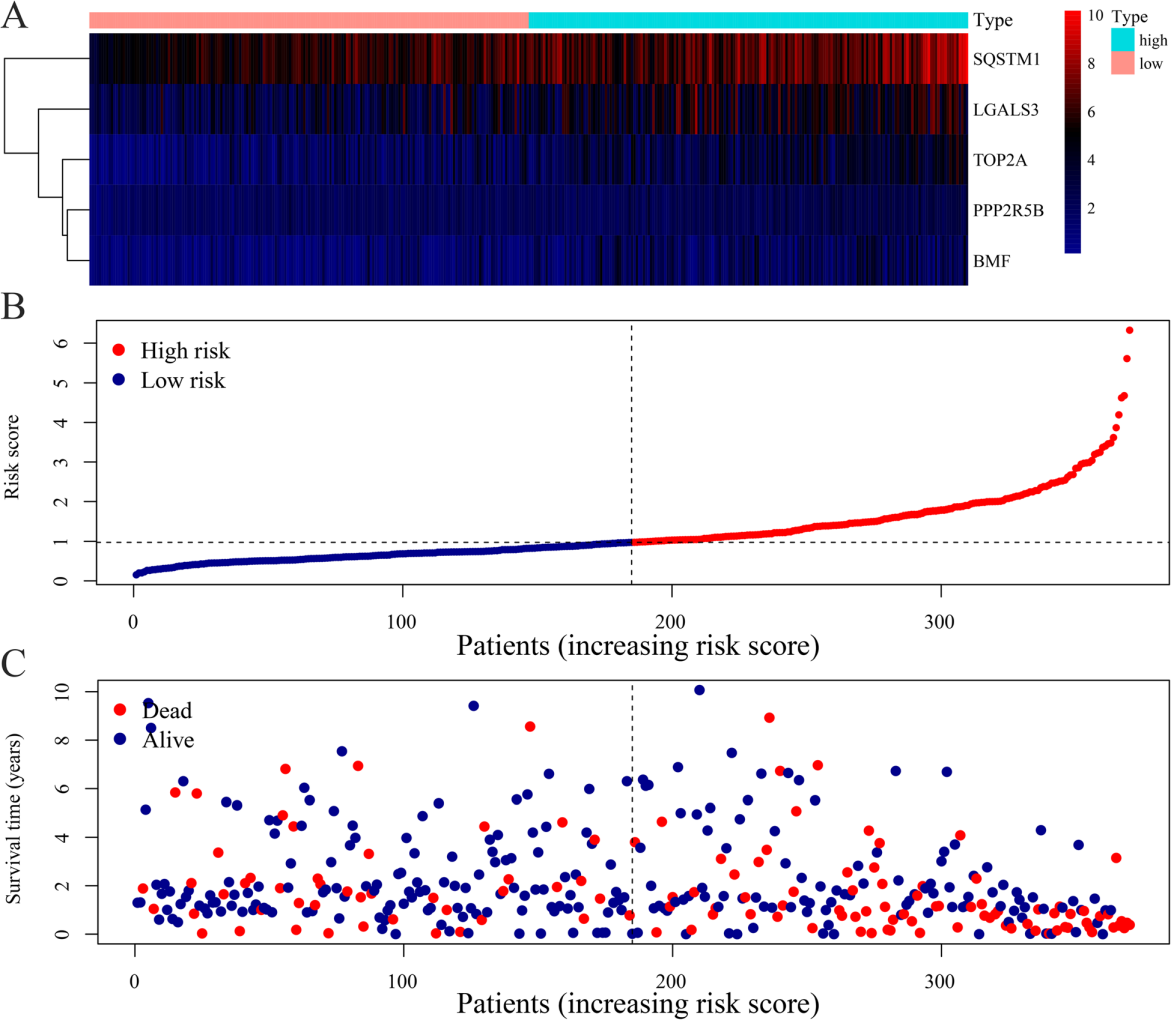
OS prediction in HCC patients by using the risk score which was established by 5 screened ARGs. **a** Expression heatmap of 5 prognostic ARGs in high and low-risk groups. **b** Risk score distribution in HCC patients. **c** Overall survival and survival status of HCC patients in the TCGA database

We combined the expression levels of ARGs and the regression coefficients of multiple Cox regression analyses to establish a risk scoring formula (Table 1). Risk score = (0.385327 × Expression value of PPP2R5B) + (0.2787 × Expression value of SQSYM1) + (0.152062 × Expression value of TOP2A) + (0.172177 × Expression value of BMF) + (0.110211 × Expression value of LGALS3). All genes had a positive coefficient, implying that elevated expression levels of the identified genes were negatively correlated with prognosis. After calculating the risk score in HCC patients, the median risk score was used as a cutoff, and these patients were assigned into high- and low-risk groups (Fig. 5). The plotted heatmap of the 5 ARGs expression levels showed that patients in the same group had distinct expression levels (Fig. 5a). Patient’s scores were ranked in ascending order (Fig. 5b) and their survival time presented as a scatterplot (Fig. 5c). Low-risk patients had a longer survival time and higher survival rates than high-risk patients.

**Table 1:** Multivariate Cox regression results of prognosis-related ARGs in HCC

Gene ID	Coefficient	HR	HR.95L	HR.95H	*p* value
PPP2R5B	0.3853266	1.4700944	1.0225253	2.1135688	0.0375054
SQSTM1	0.2786996	1.3214104	1.1163784	1.5640981	0.0011966
TOP2A	0.1520620	1.1642324	1.0033461	1.3509168	0.0450713
BMF	0.1721765	1.1878875	0.9781174	1.4426455	0.0824262

*ARGs*, apoptosis-related genes; *HCC*, hepatocellular carcinoma

**Fig. 6:**
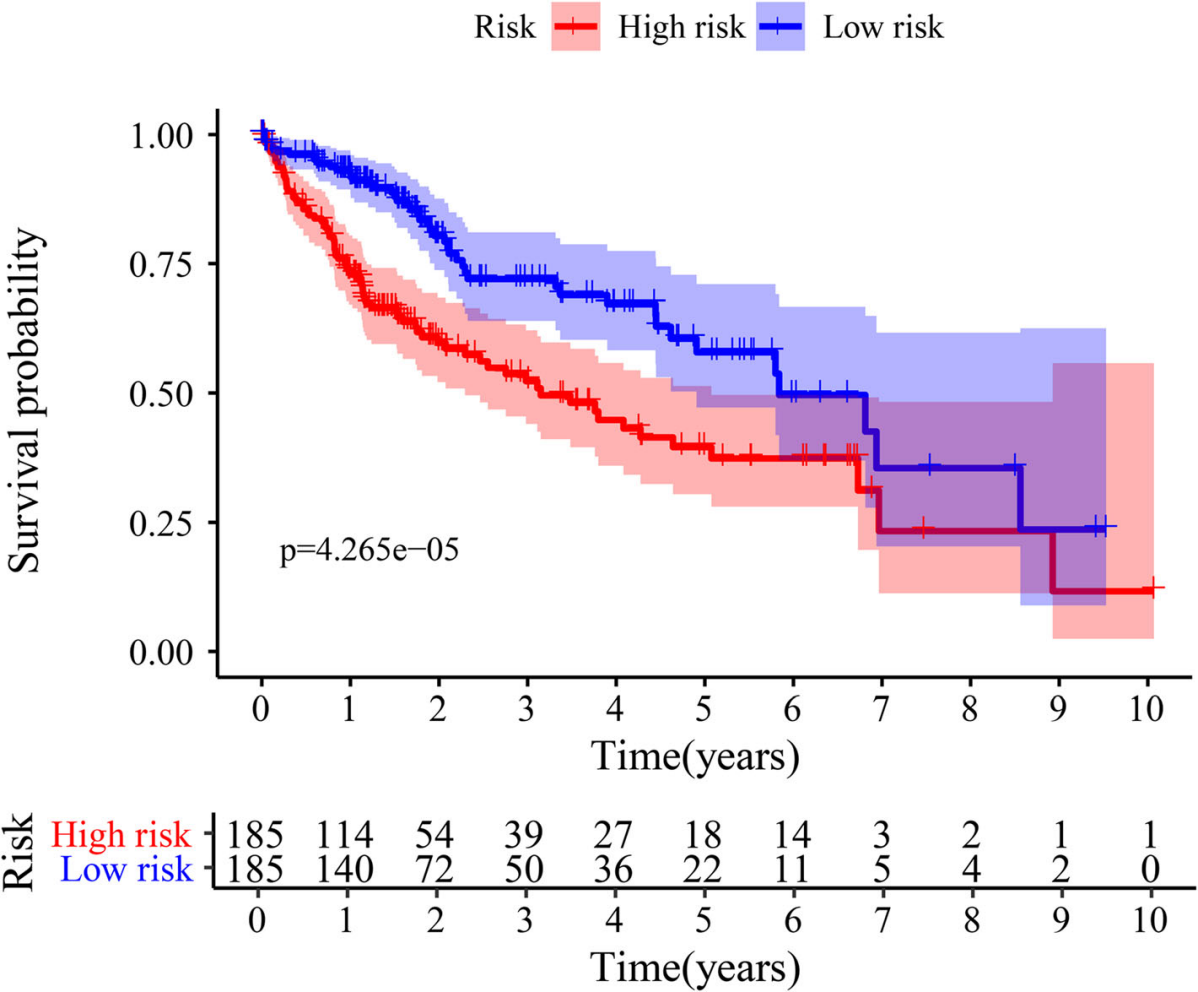
Compared with the high-risk groups, patients in low-risk groups have significantly longer OS outcomes

Survival analyses of high- and low-risk groups were performed to show the correlations between risk scores and the prognosis of patients. First, we evaluated the prognostic significance of the risk scores using K-M curves and the log-rank method as shown in Fig. 6. Compared with the high-risk group, patients in the low-risk group had a significantly high 2-year or 5-year survival probability (*p* < 0.0001; Fig. 6) revealing a worse prognosis for the high-risk group.

### Independent prediction of HCC prognosis by risk score

**Fig. 7:**
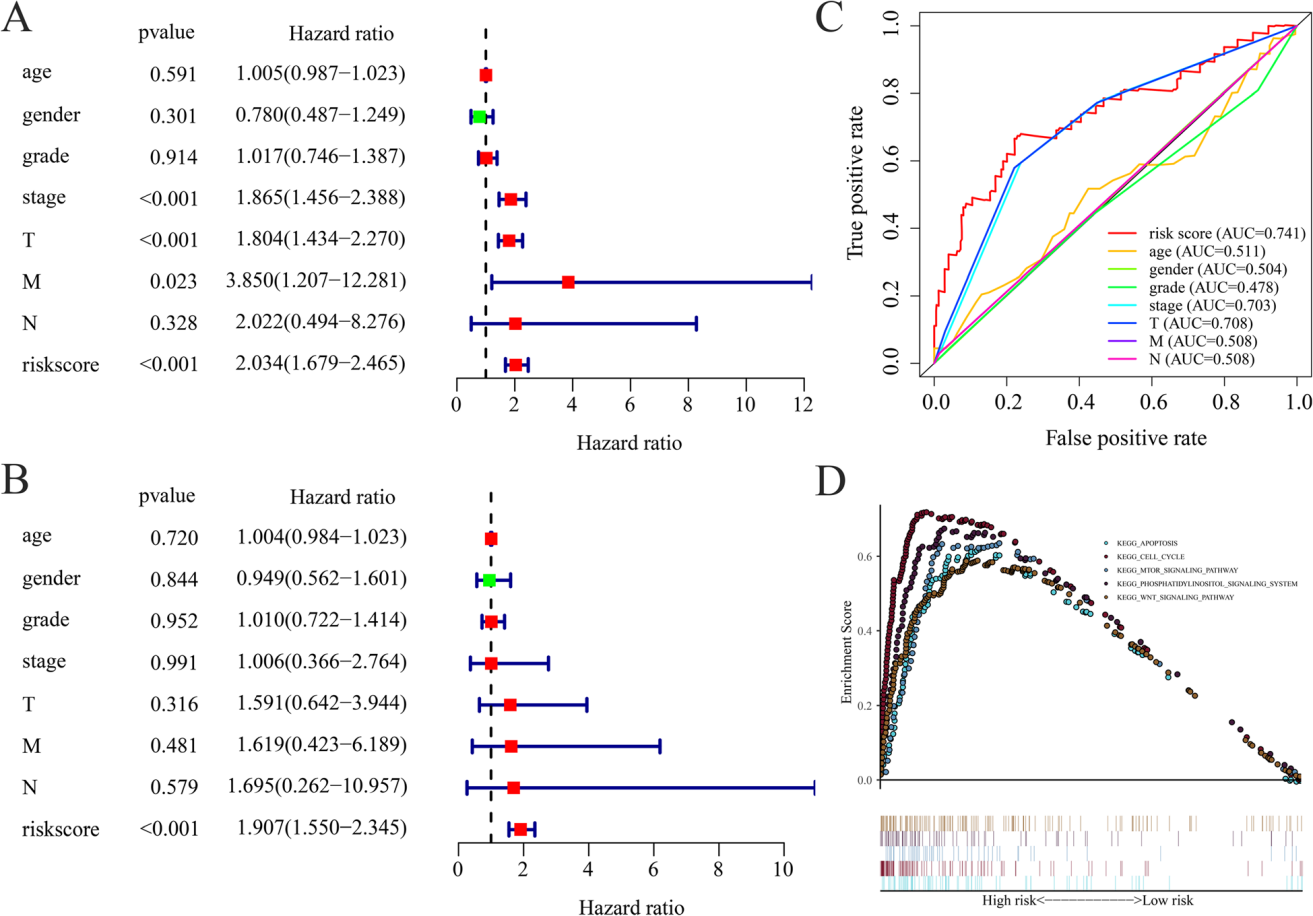
**a** Prognostic effect analysis of risk score and clinical features in HCC with univariate Cox regression analysis. **b** Independent prognostic effect analysis of risk score and traditional prognostic clinical features in HCC with multivariate Cox regression analysis. **c** The ROC analysis of the risk score and other prognostic clinical features in HCC. **d** Pathways that the 5 screened ARGs are enriched in according to KEGG analysis

Both univariate and multivariate Cox regression analyses were used to compare the predictive value of the prognostic model and other clinical prognostic variables. The risk score was established as an independent prognostic predictor for HCC patients (Fig. 7a and b). However, clinical variables were found not to be independent prognostic predictors (*p* > 0.05; Fig. 7b). Moreover, the ROC curve was plotted to validate the predictive value of the risk score (Fig. 7c). The AUC of the risk score was 0.741 as the highest value, compared with that of the clinical variables, implying that the risk score had a higher predictive value than the clinical variables.

### KEGG enrichment analysis between different risk patients

KEGG analysis was used to evaluate to explore the potential biological pathways that were associated with 5 ARGs. The 5 majorly enriched pathways were apoptosis, cell cycle, mechanistic target of rapamycin kinase (mTOR) signaling, WNT signaling, and phosphatidylinositol signaling systems (Fig. 7d).

### Construction of a nomogram to predict the prognosis of HCC patients

**Fig. 8:**
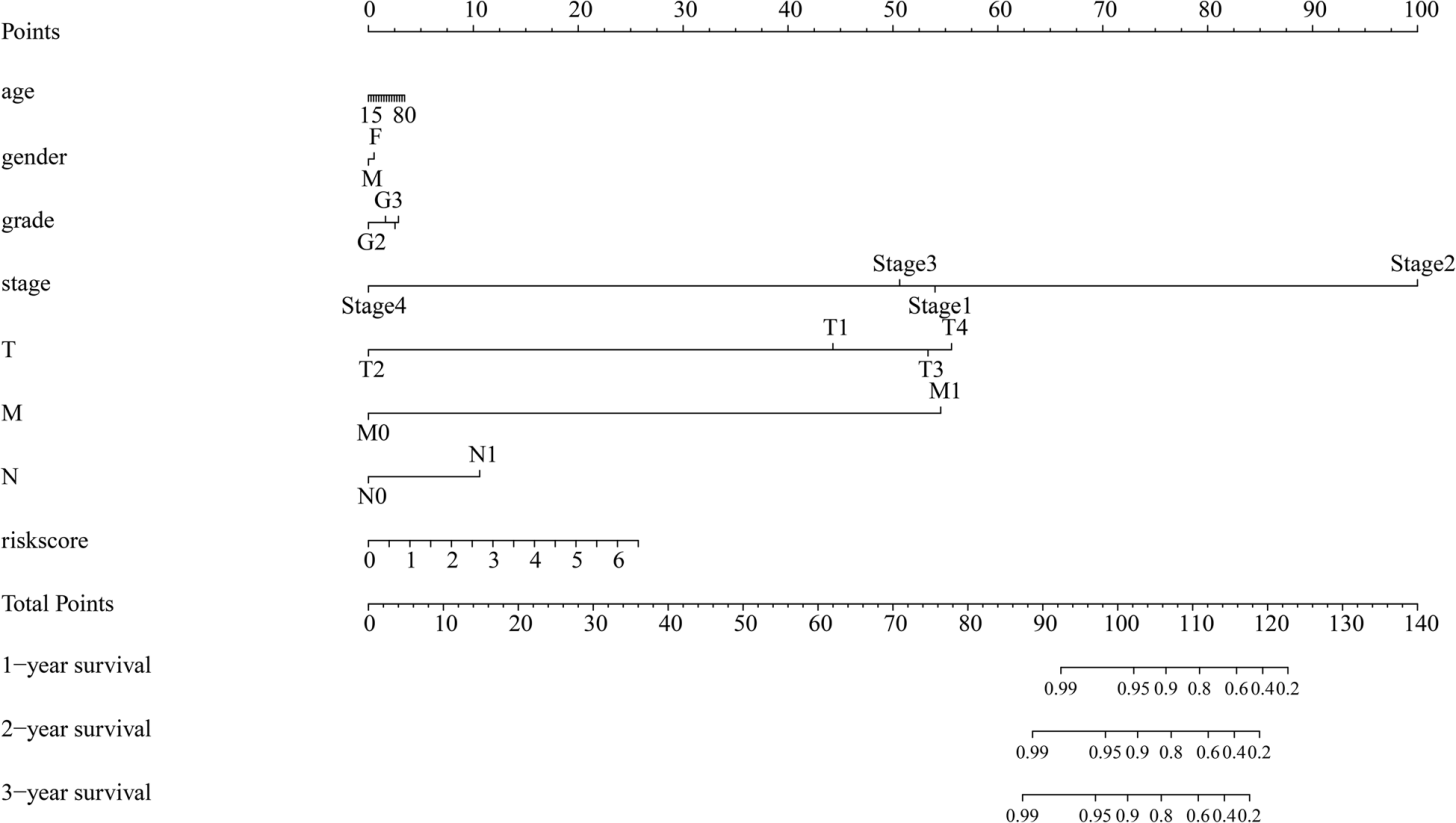
Prediction of the 1-, 2-, and 3-year OS of HCC patients with the nomogram

To apply the risk score in predicting the prognosis of HCC patients, we combined the risk score with the corresponding clinical variables to construct a nomogram to predict the OS of patients at 1, 2, and 3 years. Based on the risk scores and clinical variables, an average point for a patient can be established and extrapolated to determine the 1-, 2-, and 3-year OS (Fig. 8).

### The correlations between risk scores/ 5 ARGs and clinical variables

**Fig. 9:**
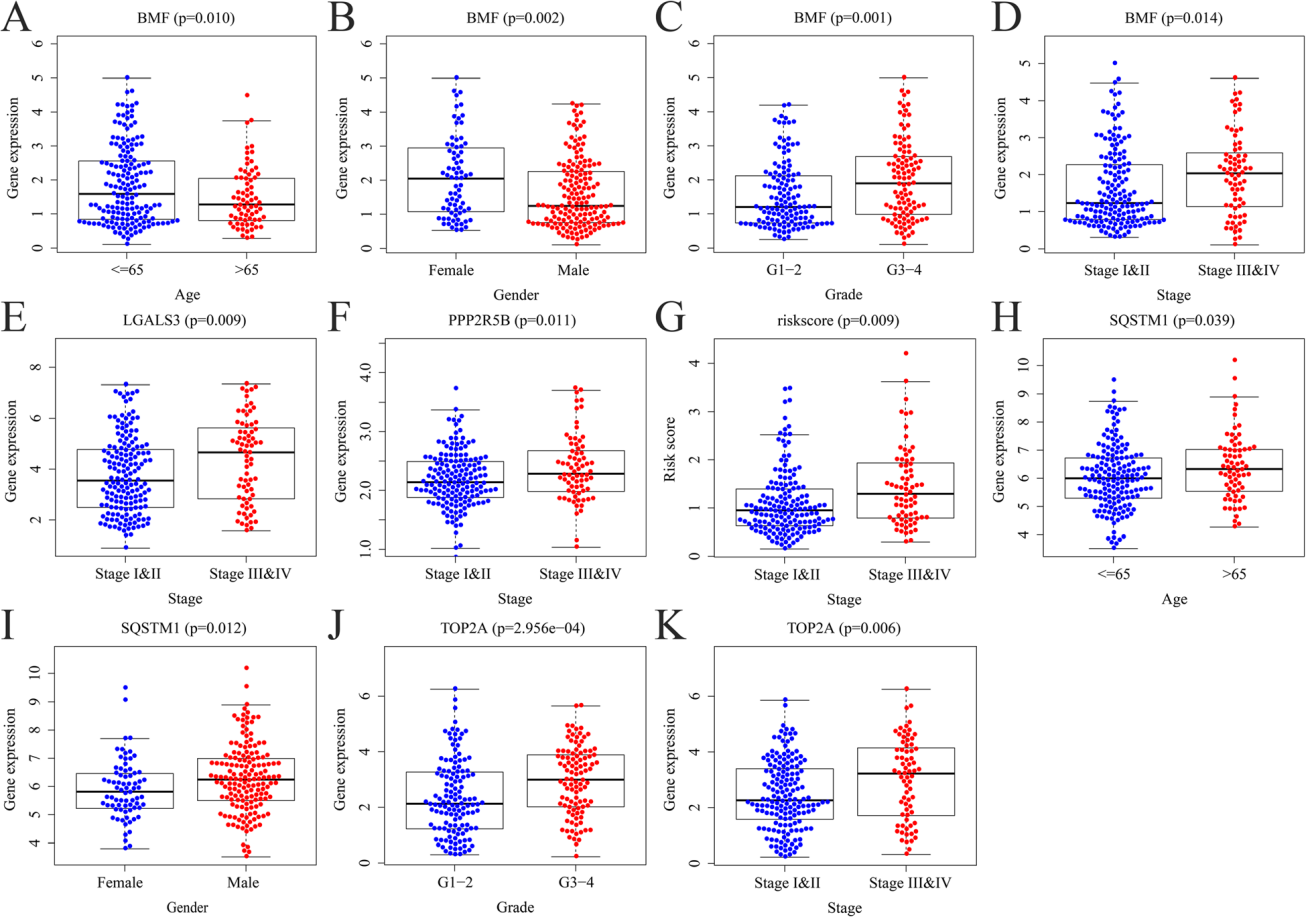
Associations between the 5 significant ARGs and clinical features. **a–d** Association between BMF expression level and age, gender, grade, and stage. **e** Association between LGALS3 expression level and pathology stage. **f** Association between PPP2R5B expression level and pathology stage. **g** Risk score and age. **h** and **i** Association between SQSTM1 expression level, age, and gender. **j** and **k** Association between TOP2A expression level, grade, and pathology stage

Based on the gene expression and corresponding clinical data obtained from the TCGA database, we analyzed the correlations between these clinical variables and risk scores of the 5 ARGs. Risk scores were correlated with tumor stage; BMF was associated with age, gender, grade, and stage; PPP2R5B and LGALS3 were correlated with stage; SQSTM1 was associated with age and gender; while TOP2A was correlated with grade and stage (Fig. 9).

## Discussion

HCC is the most common malignancy that is associated with a high recurrence rate and poor prognosis [19, 20]. Several limitations have been attributed to the late diagnosis and treatment of HCC [21]. Therefore, identification of prognostic-related biomarkers and the development of a prognostic model is of critical importance for HCC patients. Currently, bioinformatics analyses have been shown to play an increasingly significant role in identifying therapeutic targets for the diagnosis, treatment, and prognosis of numerous tumors [22]. Apoptosis plays a crucial role in liver tumor development and regeneration [12, 23]. Dysregulated apoptosis leads to the occurrence and development of liver tumors [24].

In this study, we developed a novel prognostic-predictive model for HCC based on ARGs expression. Based on the list of 161 ARGs from the GSEA and the data from the TCGA database, 43 significantly upregulated ARGs and 8 significantly downregulated ARGs in HCC samples were screened out. GO enrichment analysis revealed that these 51 genes were enriched in the pathways associated with cellular apoptosis, while KEGG analysis revealed that the 51 genes were enriched in MAPK, P53, TNF, and PI3K-Akt signaling pathways. Among the 51 genes we screened, several genes have been reported to be related to the prognosis of HCC, such as apoptosis regulator BCL2 associated X (BAX) [25], SQSTM1 [26], and CDK2 [27]. Therefore, it is feasible to evaluate a prognostic model with these genes. Besides, 5 prognosis-related ARGs (PPP2R5B, SQSTM1, TOP2A, BMF, and LGALS3) were identified. Elevated expression levels of these 5 ARGs were negatively correlated with prognosis. Based on this, we designed a risk model, and it was considered as an independent prognostic model of HCC according to the results of multivariate regression analysis. The predictive value of the model was confirmed by the K-M and ROC curves, which showed the highest prognostic predictive power of the model when combined with other clinical features of HCC patients. Therefore, by combining risk scores and clinical features as the basis for developing the nomogram, its efficiency in predicting the prognosis of HCC patients was enhanced.

The potential impact of these 5 hub ARGs on the progression of HCC were established. For example, TOP2A was identified as a potential biomarker for cancer therapy in ovarian cancer, colon cancer, pancreatic adenocarcinoma, and HCC [9, 22, 28, 29]. However, TOP2A was upregulated in HCC [30]. Overexpressed TOP2A in HCC leads to a worse prognosis, and its inhibitors have a potential therapeutic effect in HCC patients [31]. Autophagy was demonstrated to suppress spontaneous tumorigenesis in the liver [32]. SQSTM1, an autophagy-related protein, participates in cell survival, growth, and death through several pathways and degrades during autophagy [33, 34]. Overexpression of SQSTM1 gene, or abnormal aggregation and phosphorylation of SQSTM1 lead to disorder of glucose and glutamine metabolism and promote tumor development in HCV-positive HCC through persistent activation of nuclear factor erythroid 2-related factor 2 (Nrf2) [35]. Moreover, animal experiments showed that SQSTM1 was necessary for hepatocarcinogenesis in mice [26]. BMF, one of the Bcl-2 family members, promotes apoptosis by inactivating pro-survival Bcl-2-like proteins through the BH3 domain following its activation by stress signals [36]. Gramantieri et al. and Xie et al. [37, 38] revealed a close relationship between BMF and activated caspase-3. Moreover, they found that miR-221 inhibits apoptosis by targeting BMF in HCC and ovarian cancer cells. Besides, BMF inhibition promotes survival outcomes in multiple myeloma patients [39]. LGALS3 plays a significant role in the progression and metastasis of colon cancer, acute myeloid leukemia, melanoma, and pituitary tumors [40–43]. In addition, LGALS3 enhances HCC cell tumorigenesis and metastasis through the β-catenin signaling [44]. A series of studies have demonstrated that Galectin-3 can severe as a biomarker for prognosis predicting of HCC patients which are the same as our results [45–47]. PPP2R5B (B56β) as the regulatory subunit of PP2A is involved in cell growth, survival, and metabolism [48]. It has been reported that deleted PPP2R5B gene induced sensitivity to paclitaxel in cervical cancer, and this sensitivity change was supposed to be associated with apoptosis [49]. PPP2R5B mutations have been postulated to cause human overgrowth [50], though its role in HCC progression has not been elucidated.

In summary, we established a 5-gene risk model and constructed a nomogram for predicting HCC outcomes in clinical practice. Moreover, we presented an advanced survival prediction tool for HCC patients, and revealed the association between ARGs and HCC that can further be confirmed by corresponding experimental studies.

## Supplementary Information

**Additional file 1: Supplementary Table 1.** A list of the apoptosis-related 161 genes in GSEA

## Data Availability

The mRNA expression data and clinical information of HCC patients were obtained from the TCGA database (https://cancergenome.nih.gov/).
