# Supplementary material for: A prognostic model for hepatocellular carcinoma based on apoptosis-related genes
Source: World J Surg Oncol. 2021 Mar 12;19:70. doi: 10.1186/s12957-021-02175-9 (PMC7955636; doi:10.1186/s12957-021-02175-9)
Supplement: Supplementary file 1 — Additional file 1: Supplementary Table 1. A list of the apoptosis-related 161 genes in GSEA [file 12957_2021_2175_MOESM1_ESM.docx]

Supplementary table 1. A list of the apoptosis-related 161 genes in GSEA.

| HALLMARK_APOPTOSIS | | | | |
| --- | --- | --- | --- | --- |
| ADD1 | CCNA1 | ERBB2 | IL18 | PTK2 |
| AIFM3 | CCND1 | ERBB3 | IL1A | RARA |
| ANKH | CCND2 | EREG | IL1B | RELA |
| ANXA1 | CD14 | ETF1 | IL6 | RETSAT |
| APP | CD2 | F2 | IRF1 | RHOB |
| ATF3 | CD38 | F2R | ISG20 | RHOT2 |
| AVPR1A | CD44 | FAS | JUN | RNASEL |
| BAX | CD69 | FASLG | KRT18 | ROCK1 |
| BCAP31 | CDC25B | FDXR | LEF1 | SAT1 |
| BCL10 | CDK2 | FEZ1 | LGALS3 | SATB1 |
| BCL2L1 | CDKN1A | GADD45A | LMNA | SC5D |
| BCL2L10 | CDKN1B | GADD45B | LUM | SLC20A1 |
| BCL2L11 | CFLAR | GCH1 | MADD | SMAD7 |
| BCL2L2 | CLU | GNA15 | MCL1 | SOD1 |
| BGN | CREBBP | GPX1 | MGMT | SOD2 |
| BID | CTH | GPX3 | MMP2 | SPTAN1 |
| BIK | CTNNB1 | GPX4 | NEDD9 | SQSTM1 |
| BIRC3 | CYLD | GSN | NEFH | TAP1 |
| BMF | DAP | GSR | PAK1 | TGFB2 |
| BMP2 | DAP3 | GSTM1 | PDCD4 | TGFBR3 |
| BNIP3L | DCN | GUCY2D | PDGFRB | TIMP1 |
| BRCA1 | DDIT3 | H1-0 | PEA15 | TIMP2 |
| BTG2 | DFFA | HGF | PLAT | TIMP3 |
| BTG3 | DIABLO | HMGB2 | PLCB2 | TNF |
| CASP1 | DNAJA1 | HMOX1 | PLPPR4 | TNFRSF12A |
| CASP2 | DNAJC3 | HSPB1 | PMAIP1 | TNFSF10 |
| CASP3 | DNM1L | IER3 | PPP2R5B | TOP2A |
| CASP4 | DPYD | IFITM3 | PPP3R1 | TSPO |
| CASP6 | EBP | IFNB1 | PPT1 | TXNIP |
| CASP7 | EGR3 | IFNGR1 | PRF1 | VDAC2 |
| CASP8 | EMP1 | IGF2R | PSEN1 | WEE1 |
| CASP9  CAV1 | ENO2 | IGFBP6 | PSEN2 | XIAP |
| GSEA, gene set enrichment analysis. | | | | |
